# Bio-inspired colorimetric film based on hygroscopic coloration of longhorn beetles (*Tmesisternus isabellae*)

**DOI:** 10.1038/srep44927

**Published:** 2017-03-21

**Authors:** Han-bok Seo, Seung-Yop Lee

**Affiliations:** 1 Department of Mechanical Engineering, Sogang University, 35 Baekbeom-ro, Mapo-gu, Seoul 04107, Korea; 2 Department of Biomedical Engineering, Sogang University, 35 Baekbeom-ro, Mapo-gu, Seoul 04107, Korea

## Abstract

Structure-dependent colour is caused by the interaction of light with photonic crystal structures rather than pigments. The elytra of longhorn beetles *Tmesisternus isabellae* appear to be iridescent green in a dry state and turn to red when exposed to humidity. Based on the hygroscopic colouration of the longhorn beetle, we have developed centimeter-scale colorimetric opal films using a novel self-assembly method. The micro-channel assisted assembly technique adopts both natural evaporation and rotational forced drying, enhancing the surface binding of silica particles and the packing density by reducing the lattice constant and structural defects. The fabricated large-scale photonic film changes its structural colour from green to red when exposed to water vapour, similarly to the colorimetric feature of the longhorn beetle. The humidity-dependent colour change of the opal film is shown to be reversible and durable over five-hundred cycles of wetting and drying.

Structural colours arise from the physical interaction of visible light with periodic structures rather than pigments or dyes. Structural colouration in nature^1^,^2^,^3^,^4^, such as butterfly wings, beetle cuticles, fish scales, and peacock feathers, has attracted considerable attention in various fields including artificial colourations and optical sensing devices^3^,^4^,^5^. Photonic crystal structures responding to environmental changes through visual colour change can be used as low-cost colorimetric sensors without the requirement for external power sources and read-out systems. Various colorimetric sensors have been developed to detect visual changes in their structural colours induced by vapour, solvent, ion, pH, temperature, pressure, and biological materials^6^,^7^,^8^,^9^,^10^,^11^, indicating the potential in wide applications.

The optical properties of colorimetric photonic crystals can be changed by various external stimuli through the manipulation of refractive index, lattice constant, crystal symmetry, or orientation. The molecular orientation of liquid crystals can be controlled by an external electric field or exposure to a change in temperature, and therefore the structural colours of opals or inverse opals infiltrated with liquid crystals become tunable^11^,^12^,^13^. A rich variety of responsive photonic materials and liquid crystals with tunable structural colours have been developed for the applications of optical devices, colour displays and imaging systems^13^,^14^,^15^ to manipulate optical signals via the modulation of external stimuli, as well as colorimetric sensors to detect the variation in stimuli.

Humidity is important parameter in various applications, including meteorology, agriculture, process control, medical monitoring, and home appliances^16^,^17^. Some insects show visible colour changes following environmental changes or excitation. The tortoise beetle *Charidotella egregia* changes from golden in rest state to red when disturbed^18^. The cuticles of the beetle *Dynastes hercules* appear khaki-green in a dry atmosphere and turn into black under high humidity levels^19^. The male beetle *Hoplia coerulea* exhibits iridescent blue-violet colour which turns to emerald green when the elytron is put in contact with water^20^. The longhorn beetle *Tmesisternus isabellae* is also known to alter the structural colours of its elytra from golden to red exposed to humidity^21^.

A few humidity sensors based on colorimetric photonic crystals have been reported to show visible colour changes to relative humidity. Photonic crystal hydrogels^22^,^23^,^24^ or responsive materials^25^,^26^,^27^ have been used for colorimetric humidity detection, but their sensing mechanism mainly depends on the amount of expansion in photonic structures according the absorption level of environmental humidity. A colorimetric humidity sensor^28^ was developed to mimic the refractive-index mediated colour change of the beetle *Dynastes hercules* based on inverse opal photonic crystals, and the self-assembled film sensor produced hygroscopic colour-change from blue-green to red without dimensional changes by swelling. Humidity-dependent switching from transparency to coloration was also developed using mesoporous layered photonic crystals based on the refractive index variation^29^.

Biological photonic crystals of the colorimetric insects have strong mechanical durability and controllable wettability, as well as incomparable optical features of structural colouration originating from their highly-ordered periodic structures^9^. To mimic biological photonic structures, self-assembly techniques of colloidal spheres have become promising approaches because of their effectiveness, simplicity and low-cost fabrication of three-dimensional photonic structures^30^,^31^. Self-assembly methods to produce three-dimensionally ordered arrays of monodisperse spheres (colloidal crystals or synthetic opals) as well as inverse replicates of opals, have been studied intensively^11^,^31^,^32^. However, structural defects and cracks are intrinsic problems in crystallization processes and can seriously limit quality and applicability of colloidal crystals^32^. Inverse opal photonic crystals have been used to implement colorimetric humidity sensors because of relatively high reflection shifting by water infiltration. However, inverse opal structures of stimulus-responsive polymers induce structural inhomogeneity, resulting in low mechanical strength^33^. Moreover, inverse opal arrays require an additional process to form mesoporous structures from the opal frame of colloidal crystals.

The inverse-opal colorimetric humidity sensor^28^ becomes fragile after repeatable cycles of wetting and drying. Inherent structural defects and cracks, generated by the conventional self-assembly of colloidal particles, severely restrict the fabrication of large-area photonic crystals with high mechanical durability. New self-assembly methods would be required to further enhance the size and durability of humidity-responsive colloidal films.

In this study, we developed an opal-like photonic film based on colorimetric photonic structures of the longhorn beetle *Tmesisternus isabellae*, showing a similar reversible colour change in response to humidity using self-assembled silica particles. Structural defects and low durability inherent in self-assembled opals were overcome using a novel fabrication technique based on the micro-channel assisted vertical deposition and the two-stage evaporation process of natural and rotational dryings.

## Results and Discussions

### Photonic structures of the longhorn beetle

**Figure 1:**
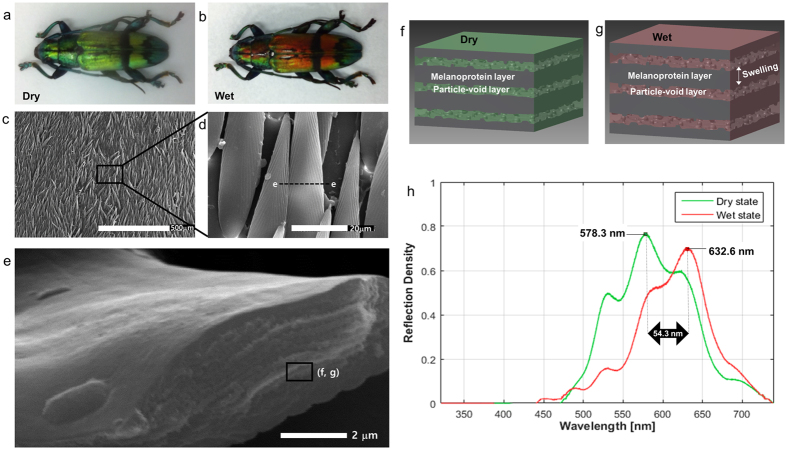
Structural colour images and photonic crystal layers of the longhorn beetle *Tmesisternus isabellae*. (**a**) Golden-green in the dry to (**b**) red in the wet state. (**c**) SEM images of the elytra show numerous long and flat scales. (**d**) Close-up view of a scale with the approximate size of 150 × 15 × 4 μm (length × width × thickness). (**e**) SEM image of transverse cross-section of a scale in dry state. (**f,g**) Conceptual illustrations from the SEM image and previous publication^21^ to describe the colorimetric photonic structure of a beetle scale consisting of two alternating layers: the homogenous melanoprotein layer and nanoparticle-void mixed one. In the wet state, only melanoprotein layers swell by water absorption, and air voids in the mixed layers are filled with water. (**h**) Measured reflection spectra of the coloured region of the beetle elytra under normal incidence were 578.3 nm and 632.6 nm in dry and wet states with a peak shift of 54.3 nm.

The structural colours of the dry and wet states of the longhorn beetle *T. isabellae* are shown in Fig. 1a,b. In the scales of the beetle, a multilayer photonic structure yields a colour switch from golden green to red exposed to humidity. As the relative humidity is increased, the golden coloured region turns red within a few minutes. The black band coloration of the elytra remains unchanged. The red coloured region recovers to the golden colour upon return to the dry state (Supplementary video 1). Thus, the humidity-induced colour change is reversible.

The magnified views of the elytral scales in the dry state were characterized by scanning electron microscopy (SEM, genesis-1000, EmCrafts) in Fig. 1c,d. The approximate length and thickness of a scale are 150 μm and 4 μm, respectively. The visible optical response to humidity is caused by multilayer structures in the interior of the scales. The transverse cross-sectional view of one scale is shown in Fig. 1e. The colorimetric photonic structure of the beetle scale consists of two alternating layers (Fig. 1f). One is a homogenous melanoprotein layer having a thickness of 100 ~ 110 nm. The other is a mixed layer consisting of melanoprotein nanoparticles and air voids. The mixed layer has a thickness of 70 ~ 80 nm with a relatively high lattice constant (the spacing between particles). The structural coloured region has a contact angle of 29.2°, facilitating water infiltration into the hydrophilic multilayers^21^. Although both melanoprotein and mixed layers absorb water, only melanoprotein layers swell noticeably after water absorption in the wet state (Fig. 1g). The air voids in the mixed layers are infiltrated with water without swelling. Therefore, hygroscopic colour change of the beetle scales occurs due to both the refractive index shift by water absorption of the mixed layers and the dimensional change by swelling of the melanoprotein layers^21^.

When a longhorn beetle *T. isabellae* is initially in a dry state and exposed to water vapour, the reflection spectra of the coloured region of elytra can be measured using a spectrometer analyser (CCS100, Thorlabs, Newtown, NJ, USA). The elytra of the beetle in the dry state show a golden green colour with the reflectance peak at 578.3 nm (Fig. 1h). The reflection peak shifts from 578.3 nm to 632.6 nm with a peak increment of 54.3 nm, causing a dramatic change in the structural colour. The measured reflection peaks are consistent with colour observation by the naked eye. The reflection spectrum band is relatively broad because the beetle scale has multilayer structures consisting of alternating high and low refractive-index layers^21^.

### Design of bio-inspired colorimetric opal films

In order to fabricate colloidal opal films to have a similar humidity-dependent colour change (green to red shift) to the longhorn beetle *T. isabellae*, we calculated the size of colloidal particles to fill in three-dimensional photonic crystals. The theoretical reflection wavelength of opal photonic crystals composed of spherical particles was approximately predicted using a modified Bragg’s equation for normal incidence, 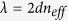. Here *λ* is the peak wavelength of reflected light, *d* = 0.816*D* is the interlayer spacing in the [1 1 1] direction, *D* represents the mean diameter of colloidal particles, and 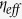
is the effective refractive index of the sample. The effective refractive index of a two-phase structure can be estimated as^28^,^34^, 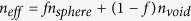. Here *f* = 0.74 is the fraction of the colloidal particles for an ideal face-centred cubic package. *n*_*sphere*_ and *n*_*void*_ are the refractive indices of the colloidal particles and air void, respectively.

In this study, we use silica particles with a refractive index of *n*_*sphere*_* = *1.55. In the dry state, *n*_*void*_ = *n*_*air*_ = 1. In the wet state, water infiltrates into the air void and the refractive index becomes *n*_*void*_ = *n*_*water*_ = 1.333. Using the given parameters, we calculated the diameter of silica particles for the bio-inspired humidity sensor using the Bragg’s equation. At *D* = 252 nm, the theoretical reflection wavelength of opal photonic crystals is *λ* = 578 nm (green colour) in the dry state. When water penetrates into the air voids in the wet state, the theoretical reflection peak shifts to *λ* = 615 nm (red colour). The theoretical photonic band-gap shift due to only water penetration into the opal structures without swelling can be calculated asΔ*λ* = 615–578 = 37 nm. Therefore, we expected that opal photonic structures using silica particles with a diameter of 252 nm would generate bio-inspired hygrochromic coloration (from green to red), similarly to tunable color of the longhorn beetle.

### Optical and mechanical features of self-assembled opal films

**Figure 2:**
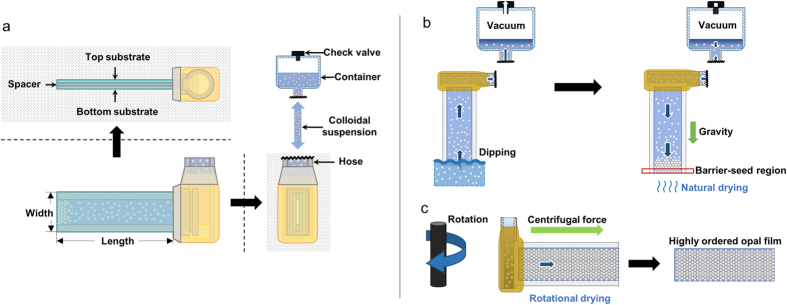
Fabrication procedures of opal film using a rotating self-assembly technique. (**a**) Preparation of experiments by making a micro-channel system consisting of two glass substrates and Parafilm spacer for the proposed self-assembly fabrication. (**b**) The micro-channel was dipped into colloidal suspensions and then naturally dried for 1 h. (**c**) By rotating the channel system and detaching the top substrate, an opal photonic film with the high packing density and mechanical durability was fabricated.

A novel self-assembled process for fabricating colloidal crystals using a micro-channel assisted dip-coating and a rotational drying method was proposed by the authors^35^. The rotating self-assembly technique for producing the colorimetric opal film is summarized in Fig. 2. Besides predominant point and line defects within self-assembled colloidal crystals, macroscopic cracks inevitably evolve during the drying of the colloidal particles on rigid substrates due to compressive stresses perpendicular and tensile stresses parallel to the plane of the support material^36^,^37^. In order to reduce defects and cracks in opal films, the proposed self-assembly adopted a two-stage drying process of natural evaporation (Fig. 2b) and rotation-induced forced drying (Fig. 2c).

**Figure 3:**
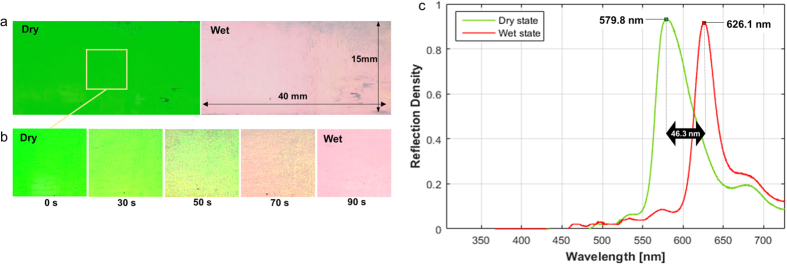
Photographs and optical features of the fabricated film exposed to water vapour. (**a**) Green in dry state and turn to red in fully wet state. (**b**) Colour change from green to red according to the exposure time of water vapour. (**c**) Measured reflection spectra of the fabricated opal film under normal incidences were 579.8 nm and 626.1 nm in dry and wet states with a peak shift of 46.3 nm.

Figure 3a shows the fabricated colorimetric opal film using the self-assembled silica particles with a diameter of 252 nm. The fabricated opal film was 15 mm × 40 mm × 100 μm in size. The structural green colour was clearly displayed in the dry state. When the film was exposed to water vapour, its colour changed to red in a few minutes. Figure 3b shows the structural colour change of the film according to the exposure time to water vapour. The entire film became completely red after 90 s. The red colour changed to the original green colour when the photonic film was exposed to air.

On the other hand, the rotational drying process does not only enhance the structural uniformity and durability of the opal film, but it also reduces dramatically the time required for colloidal crystallization, compared to conventional evaporation methods. An average of approximately 150 min is required to fabricate opal structures with the size of a standard glass substrate using this method. The manufacturing time of the photonic crystal structures depends mainly on the thickness of the micro-channel and increases with decreasing thickness.

The spectrum wavelengths of the fabricated film in the dry and wet states are shown in Fig. 3c. After perpendicularly generating a white light source to the surface of the film, the reflecting light was collected to analyse optical spectra. The reflection peak in the dry state was observed at a wavelength of 579.8 nm, which shifted to 626.1 nm in the wet state. The measured reflection shift was Δ*λ* = 626.1–579.8 = 46.3 nm. The experimental reflection peaks using silica particles with a diameter of approximately 250 nm agree with the theoretical values (578 nm and 615 nm) in the dry and wet states and a peak shift of 37 nm. The reflection spectra of the fabricated film in the dry and wet states are similar to those of the longhorn beetle *T. isabellae*. The opal film, consisting of monosized silica spheres, has much narrower reflection peaks compared to complex multilayer structures of the longhorn beetle. On the other hand, a higher reflection peak shift (Δ*λ* = 54 nm) of the longhorn beetle (Fig. 1h) was observed. It is because the colour change of the beetle elytra is due to the combined effect of the refractive index shift by water infiltration and swelling of the melanoprotein layers^21^. Using self-assembly with hard spheres, the particle size dispersity and the defect density deteriorate the optical properties of monodispersed photonic crystals^38^. The reduced defect density is directly reflected in remarkably sharp and tunable Bragg peaks in the optical absorption. Therefore, relatively sharp reflection peaks of the opal film (Fig. 3c) also indicate the low size dispersity of colloidal particles and low defects in photonic crystals.

**Figure 4:**
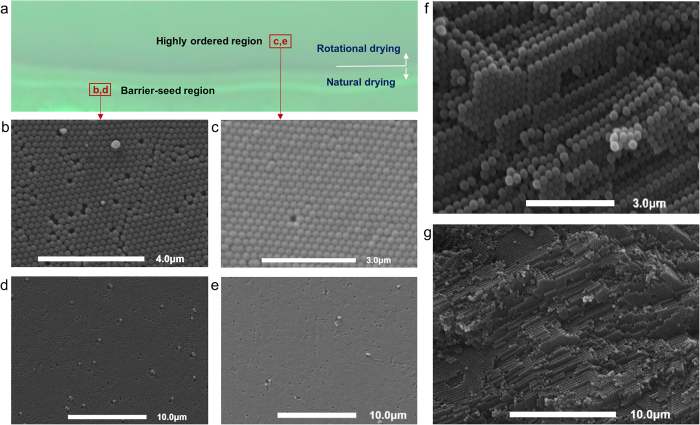
Structural densification and uniformity of the opal film fabricated by natural and rotational dryings. (**a**) Magnified photo view of the fabricated opal film near the barrier-seed region. (**b,d**) Top-down SEM images in two magnifications of the barrier-seed region show that loose-packed colloidal crystallization was constructed by only natural evaporation with a lot of line and point defects. (**c**,**e**) SEM images of the highly ordered region assembled by rotational drying display structural densification and uniformity of colloidal particles with low defects. (**f,g**) Cross-sectional SEM images of the highly ordered region confirm that the closed-packed crystallization has FCC structures.

Figure 4a shows a magnified photograph of the fabricated opal film near an interface between the barrier-seed region and the rotation-induced crystallization. There existed many discontinuous layers in the barrier-seed region, which was constructed by natural evaporation and gravitational sedimentation of colloidal particles. This structural non-uniformity was mainly due to inconsistent convection and evaporation, induced by environmental variations such as temperature and humidity during the relatively long-time natural-drying process. Figure 4b,d display scanning electron microscope (SEM, genesis-1000, EmCrafts) images in two magnifications of the barrier-seed region. The natural evaporation process constructed loose-packed crystallization with lack of structural densification and uniformity. The crystal structure included many line defects on discontinuous interfaces of the loose-packed colloidal assembly, as well as point defects. The barrier-seed region, which was constructed at the channel tip during natural drying, prevented the leakage of suspension during rotational drying. Moreover, it acted as seed layers by providing a lattice frame for the self-assembly of colloidal spheres during rotational drying.

Figures 4c,e show SEM images under two magnification factors of the highly ordered region, which was constructed by rotational drying. Since the surface binding of colloidal particles was enhanced, close-packed structures were assembled with high densification. There were no line defects or cracks in the opal film except for point defects. Some dot-like defects were present because of the size dispersity or geometric shape of the silica particles. The local defects that formed in the fabrication process grow extensively in disturbance or repeated use, and therefore structural durability of photonic crystals could be degraded. Since colloidal particles pack together without chemical bonds, the loose-packing of silica particles by natural drying include could be easily disassembled due to defect growth and crack propagation by exposure to repeatable humidfication. However, the rotational drying technique intensified colloidal aggregation and densification, enhancing structural durability to external disturbances. The quality of the nano-sized spheres used is also crucial in order to obtain crystalline order in colloidal crystals. Two main limiting factors for the fabrication of colloidal crystals are the presence of spheres smaller than the average diameter and nonspherical particle formation^39^. It is noted that the rotational drying method can dramatically reduce defect density in crystallization of colloidal particles, compared to the naturally dried photonic crystals with the same colloidal size dispersity. The transverse cross-sectional SEM images of the close-packed region display three-dimensional face-centered cubic (FCC) structures with approximately 400 layers in the 100-μm-thick film (Fig. 4f,g). The cross-sectional views confirmed that the photonic film consisted of FCC structures rather than hexagonal close-packed (HCP) crystallization with the same packing density (0.74).

**Figure 5:**
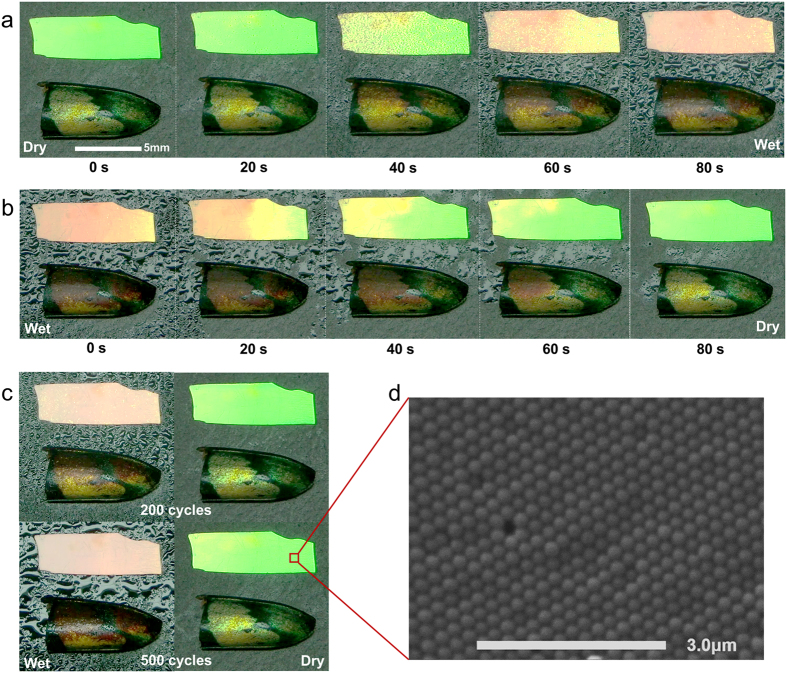
Hygrochromic comparison of the colorimetric film and the beetle elytra. (**a**) Photographs of partial pieces of the film and beetle elytra according to the exposure time of water vapour. (**b**) Photographs of reversible colour changes during natural drying show very similar hygrochromic characteristics between samples. (**c**) Colour-change images of the opal film and elytra after 200 and 500 cyclic wetting and drying experiments. (**d**) SEM image of the opal film after 500 cycles of wetting and drying confirm that the close-packed colloidal crystal has mechanical stability and durability to repetitive humidification.

To directly compare the longhorn beetle and bio-inspired colorimetric film, partial pieces of the film and beetle elytra were exposed to cyclic humidity conditions. Figures 5a,b show the colour variations of both samples over a cycle of successive wetting and drying. Both samples showed very similar colour changes according to the exposure time of water vapour, and became red when they were completely wet in the water after 80 s. Moreover, the samples recovered to their original colours after drying, demonstrating that the hygroscopic colour-change of the film sensor is reversible (Supplementary video 2). Colour-change images of the opal film and elytra were display after 200 and 500 cyclic wetting and drying experiments using a humidifier and an electric drier (Fig. 5c). The film sample showed good durability without additional defects or cracks for five-hundred cyclic wetting and drying experiments. Notably, the colorimetric opal film showed reversible and repeatable performance in humidity detection, as well as excellent mechanical stability.

Most of colloidal crystals are very fragile because colloidal particles pack together without chemical bonds and the ordered packing of the particles can be easily disassembled in water or solvents^40^. Repeatable wetting-drying disturbances on self-assembled colloidal crystals can cause defect growth and crack propagation, leading to abrupt structural failure. However, SEM image of the opal film after 500 cycles of wetting and drying confirmed that the close-packed colloidal particles had not been disassembled, having structural durability to repetitive humidification (Fig. 5d). The experimental results of the fabricated opal film proved that the proposed drying technique dramatically reduced structural defects or cracks and it also intensified colloidal aggregation without additional crosslinking agents^41^,^42^.

For the practical applications of tunable photonic crystals, it is required to manufacture large-scale photonic crystals with low defects in a highly efficient and reproducible method. Several self-assembly techniques have been proposed for large-area photonic crystals using spin coating^43^, doctor blade coating^44^, spray coating^45^, and a roll-to-roll Langmuir-Blodgett (LB)^46^ methods. However, it is still difficult to fabricate highly scalable, defect-free colloidal crystals of non-crosslinked spheres. In addition to the requirements for high scalability and low defect density, structural durability to repeatable humidification is also necessary for the hygroscopic photonic films. This proposed fabrication method, based on the micro-channel deposition and the two-stage evaporation process, could be one of practical self-assembly techniques for the colorimetric application of tunable photonic crystals.

## Conclusion

We were able to design and fabricate a centimeter-scale opal film over a using a rotational self-assembled technique, inspired by tunable structural colour of the longhorn beetle *T. isabellae*. The photonic film changed in colour from green to red when exposed to water vapour, which is similar to the hygrochromic feature of the longhorn beetle. This proposed opal film showed reversible and durable colorimetric detections over five-hundred cyclic experiments, making it a low-cost structural coloration and sensing mechanism for quantifying environmental humidity without electricity.

## Methods

### Materials and Preparations

The micro-channel consists of two tempered glass substrates, and each substrate is 25 × 75 × 2 mm (width × length × thickness) as shown in Fig. 2a. In order to form coating layers for low surface energy, a pre-polymer of PDMS and crosslinking agent were mixed in a 4:1 ratio. Next, the mixture was coated onto each substrate with a thickness of approximately 50 μm, followed by hardening for 1 h at about 90 °C in a vacuum oven. A spacer, located between the two substrates, controlled the thickness of the self-assembled photonic crystals. Parafilm (M, Bemis, Neenah, WI, USA) was used as spacer to provide the stable adhesion and removal between the two substrates. Parafilm is 4 mm × 75 mm × 200 μm. Both substrates were bonded by heating at 80 °C for 15 min in a vacuum oven. A hose was used for the colloidal transfer from a container of colloidal particles to the micro-channel. The coupling parts, consisting of sequential layers of substrate-Parafilm-hose-Parafilm-glue-epoxy, were combined and sealed to prevent leakage. The coupling parts exhibited adhesive strength to endure fluid pressure up to 300 kPa. Loss of the inner pressure in the container was prevented by a rubber stopper or one-way check valve.

### Fabrication by Rotational Self-Assembly Technique

The first step in fabricating opal photonic structures was to immerse the micro-channel into colloidal suspensions (Fig. 2b, left). The colloidal suspensions were composed of silica particles with a diameter of approximately 250 nm and concentration of approximately 20 vol% to de-ionized (DI) water solvent. The resulting immersion capillary forces dragged the colloidal particles together into the micro-channel. The suspensions flowed and filled the channel-hose-container in sequence under vacuum of the container. In the second fabrication step, the surface of the channel contacting the atmosphere was set to be vertical to the direction of gravity for natural dispersion of colloidal suspensions (Fig. 2b, right). The device was naturally dried for 1 h at a relative humidity of 25%, temperature of about 20 °C, and pressure of 1 atm. During natural dispersion, the colloidal particles moved to the outside end of the micro-channel and began to crystallize in a local area by gravitational sedimentation, forming a barrier-seed region. The barrier-seed region was approximately 3 mm in the longitudinal direction from the outside air-contacting end and had a cross-sectional area of 15 mm × 100 μm.

In the final step, centrifugal force was applied by rotating the self-assembly apparatus. As shown in Fig. 2c, the direction of centrifugal force was parallel to the longitudinal direction of the channel and vertical to the direction of the width. Thus, the rotating self-assembly method differs from the widely used spin-coating method. The opal photonic structure, formed inside the micro-channel, included water solvent in air gaps. Thus, all colloidal suspensions remaining in the hose were removed to prevent their transfer to the channel. As the self-assembly system was rotated at the speed of 380 rpm, the micro-channel was dried from the outside end which contacted the atmosphere. As the inner solvent began to be eliminated, highly ordered opal photonic structures were produced from the end of the atmosphere-contacting surface inside the micro-channel. Because Parafilm, glue, and epoxy around the hose-combined parts were vulnerable to heat, they were easily removed by heating after the rod-shaped photonic structures were formed. The top-substrate was removed by increasing the temperature over approximately 40 °C to the Parafilm used as a spacer. In this self-assembly process, unintended structural defects in the local areas could be additionally generated when the top substrate was removed after the two-stage dryings, because of the adhesion of some nanoparticles to the substrate.

## Additional Information

**How to cite this article:** Seo, H.-B. and Lee, S.-Y. Bio-inspired colorimetric film based on hygroscopic coloration of longhorn beetles (*Tmesisternus isabellae*). *Sci. Rep.* **7**, 44927; doi: 10.1038/srep44927 (2017).

**Publisher's note:** Springer Nature remains neutral with regard to jurisdictional claims in published maps and institutional affiliations.

## Supplementary Material

Supplementary Information

Supplementary Video 1

Supplementary Video 2
