# Supplementary material for: Bio-inspired colorimetric film based on hygroscopic coloration of longhorn beetles (Tmesisternus isabellae)
Source: Sci Rep. 2017 Mar 21;7:44927. doi: 10.1038/srep44927 (PMC5359605; doi:10.1038/srep44927)
Supplement: Supplementary Information [file srep44927-s1.doc]

**Supplementary Information**

Bio-inspired colorimetric film based on hygroscopic coloration of longhorn beetles (*Tmesisternus isabellae*)

Han-bok Seo^1^ & Seung-Yop Lee^1,2^

^1^Department of Mechanical Engineering, Sogang University, 35 Baekbeom-ro, Mapo-gu, Seoul 04107, Korea.

^2^Department of Biomedical Engineering, Sogang University, 35 Baekbeom-ro, Mapo-gu, Seoul 04107, Korea.

Correspondence and requests for materials should be addressed to S.L.(email: sylee@sogang.ac.kr)

**Supplementary Videos**

**Video 1**. **Colour change of the longhorn beetle *Tmesisternus isabellae* over a cycle of wetting and drying**. The structural colour of its elytra is altered from golden to red exposed to humidity and the red coloured region recovers to the golden colour upon return to the dry state.

**Video 2**. **Colour-change comparison of the bio-inspired opal film and the beetle elytra over 3 cycles of wetting and drying**. Both samples showed very similar colour changes according to the exposure time of water vapour, and became red when they were completely wet in the water after 80 s. This video is 4 times faster than it is recorded.
